# Would Renationalisation and Co-financing of the Common Agricultural Policy Be Justified?

**DOI:** 10.1007/s10272-022-1038-5

**Published:** 2022-04-08

**Authors:** Ákos Kengyel

**Affiliations:** Faculty of Economics, Dep. of World Economy, Corvinus University of Budapest, Közraktár utca 4-6, H-1093 Budapest, Hungary

**Keywords:** H77, Q18

## Abstract

During the past decades several attempts have been made to reform the Common Agricultural Policy (CAP). The article analyses the possible new directions of CAP implementation and financing of the CAP. It discusses whether renationalisation and co-financing of the CAP would be a beneficial approach to make the policy more efficient and to help restructuring not just the CAP expenditure but the whole EU budget. The author analyses the changes in light of the new regulatory frameworks to be implemented from 2023.

The Common Agricultural Policy (CAP) has been subject to changes several times. This demonstrates that the CAP is always developing to meet new economic, societal and environmental needs. Due to the changing role of European agriculture, gradual and continuous reforms are justified. Given the different situation in individual member states, many of the desirable changes can be supported more efficiently at the national level. Therefore, the gradual shift to a CAP emphasising the multifunctional role of agriculture, with a greater focus on environmentally friendly farming and maintaining the appearance of the countryside, logically raises the issue of renationalisation.

Reforms notwithstanding, the CAP is the most integrated of all EU policies and consequently takes a large share of the EU budget. The present CAP is divided into two pillars. Pillar 1 (market measures and direct payments) expenditure is fully financed by the EU budget, and Pillar 2 (rural development) spending is co-financed by member states (varying from 25% to 75% depending on the measures and region). Additional reforms are unavoidable in the future in order to reach a more efficient and effective implementation and more balanced way of financing the desirable priorities.

A political agreement was reached in June 2021 on a "fairer, greener and more flexible" CAP integrating environmental and climate policy goals in line with the Green Deal and its Farm to Fork and Biodiversity strategies (European Commission, 2021e). Finally, the reform package was approved by the European Parliament in November 2021.[1] The new CAP regulation will enter into force in 2023. Because of the new elements defined in the regulatory frameworks, it can be stated that the future CAP will represent a paradigm shift in the implementation of agricultural activities in 2023-2027. However, the proposed changes will not lead to a drastic renationalisation of the policy, especially as far as budgetary resources are concerned.

The new requirement to prepare national CAP Strategic Plans at the member state level shows a kind of shift towards renationalisation of the policy in some respect. The new delivery and implementation frameworks can help to improve the efficient use of the available budget for agriculture. On the other hand, during the negotiations on the future regulatory frameworks, member states did not take the introduction of national co-financing of the direct payments into account. Due to several considerations, the member states clearly reject these further steps towards renationalisation. However, renationalisation and co-financing of the CAP could jointly support and implement agricultural policy objectives in a more efficient and flexible way.

## Fulfilled original objectives and questioned efficiency

The Common Agricultural Policy has become one of the most regulated and most controversial policies of the EU (Lynggaard and Nedergaard, 2009; Kengyel, 2014; Kuhmonen, 2018). Agriculture has been at the heart of the European integration process since the very outset due to

**Ákos Kengyel**, Corvinus University of Budapest, Hungary.

1 See the background documents on the website of the European Parliament (2021).

food market instabilities, the disproportionate influence of food prices on inflation and a need to maintain domestic food industries for political reasons (Germond, 2019). The CAP initially sought to increase agricultural productivity in the EU and secure availability of food supplies. Originally, it was intended to be a form of protectionism to defend less competitive and more expensive European producers from cheaper products outside the EU.

The established objectives have generally been met. As a result of the CAP, agricultural productivity improved, significant technological modernisation took place, the quality of agricultural produce increased, average crop yields rose, markets became stable, supply security has been achieved and an adequate income level for agricultural workers has been reached. The funding made it possible to build agricultural infrastructure, i.e. storage capacities, transport vehicles and cold storage facilities essential for agricultural activity.

However, it should be noted, that the subsidy system has not only become incredibly expensive, but it has also created inequality between farmers (Giannakis and Bruggeman, 2015). In reality, direct payments of the CAP are somehow distorted, which means that large industrial farms get significantly more financial support than medium- or small-sized farms that are actually in need (Krezminski, 2019; Niemi and Kola, 2005). An evidence-based fitness-check of the CAP has pointed out several inconsistencies (Pe'er et al., 2017). The study concluded that the CAP has had a positive effect in supporting farm incomes, however, direct payments have created dependencies on subsidies and reduced efficiency. The study stressed that the distribution of direct payments is highly inefficient and poorly justified, and, in addition, there is no clear link between objectives and instruments.

There are several scholars who call attention to the fact that the defendants of the direct payments have found new objectives to maintain the high level of funding (Erjavec and Erjavec, 2015). The "greening strategy" proved to be successful because the subsidies can be seen as remuneration for farmers to preserve the environment and biodiversity (Heinemann, 2017; Navarro and López-Bao, 2019). Because of lacking binding requirements, greening conditions have not reached their original goals (Lakner et al., 2019) and have not been effective enough to change agricultural production (Heinemann and Weiss, 2018; Pe' er et al., 2019).

## New delivery model for the period 2023-2027

According to the future regulatory frameworks of the CAP, the EU wants to ensure CAP's ability to continue providing uniquely strong support for farming, improving rural areas and high-quality food production within the EU. The future CAP wants to play a crucial role in strengthening the efforts of European farmers to contribute to the EU's climate objectives and to protect the environment (European Commission, 2021e). The implementation of the proposed reforms will only start by January 2023, due to the long-lasting negotiations among the member states and between the Council of the EU and the European Parliament. The 2014-2020 CAP regulations continue to apply throughout the first two years of the 2021-2027 Multiannual Financial Framework (MFF).

In the period 2023-2027, the new delivery model will bring both pillars of the CAP under a single strategic planning process, allowing for complementarity and synergy between the two pillars. The legislative proposal defines the expected content and structure of the national CAP Strategic Plans, including key features such as an assessment of needs and a description of the different interventions to be used as part of a member state's overall intervention strategy. The new model will allow the member states to plan a mixture of mandatory and voluntary measures in both pillars to meet the environmental and climate objectives defined at the EU level (Bourget, 2021; Rac et al., 2020; McEldowney and Rossi, 2021).

The new green architecture will be based on enhanced conditionality and additional environmental and climate related requirements that can be achieved through the eco-schemes in Pillar 1 and environmental and climate-related measures in Pillar 2. Member states will describe eco-schemes in their CAP Strategic Plans. Eco-schemes should be key tools to deliver on the Green Deal targets (European Commission, 2021d). At least 35% of the rural development budget should be spent on environmental and climate-related measures and, as a general rule, at least 25% of the direct payments budget should be dedicated to eco-schemes, which would be voluntary but would increase farmers' income (Table 1).

The new design and implementation of the CAP can have a positive impact on the overall greening of the policy, but it will be dependent on implementation at the member state level. Concerning the increased environmental ambition, there are no guarantees of achieving the desirable objectives because the proposed safeguards are too weak. More accountability would be required during the formulation and implementation of national Strategic Plans. Several research studies call attention to flexibility, which member states often use to follow the least ambitious approach, especially for environmental and climate targets (Matthews, 2018; Rac et al., 2020). Without necessary accountability mechanisms due to the lack of quantified objectives at EU

Table 1
**Main changes in the CAP regulatory frameworks**

| | Current system | New system (2023-2027) | Main changes |
|---|---|---|---|
| Delivery and implementation | Strategic planning only in Pillar 2 (rural development programmes) | Introduction of national Strategic Plans for the entire CAP | Greater flexibility for the member states but within constraints of common objectives; supervisory role for the Commission |
| Environmental conditionality/ Green architecture | Cross compliance: conditioning of some payments on compliance with certain requirements, including environmental ones (Pillar 1 and some Pillar 2 payments) Greening requirements related to direct payments, and voluntary agri-environmental and climate measures (Pillar 2) | Enhanced conditionality replaces cross compliance and greening requirements Flexible eco-schemes (in Pillar 1) and environmental and climate-related measures (in Pillar 2) | A mixture of mandatory and voluntary measures in both pillars to meet the environmental and climate objectives defined at EU level |

Source: The author's own compilation.

level, a race to the bottom is possible (Heinemann and Weiss, 2018, 12-13).

The CAP Strategic Plans will be assessed in light of the Green Deal. According to the planned timetable, the Commission will assess whether Green Deal targets are likely to be met by 2025 and readjust Strategic Plans through Performance Review in 2026-2027. Theoretically, the indicators must be meaningful in relation to the objectives and impact indicators should be used as promptly as possible during the funding period to assess success (Schulze, 2018; Moore, 2021). Based on the new regulation, mainly output and result indicators will be used, whereas the impacts will be analysed only after the end of the funding period within the framework of ex post evaluation starting from 2028. This approach does not appear to be the most efficient way to monitor and evaluate the achieved goals.

## Declining but high budgetary transfers

The CAP has always been criticised for its large budget and for supporting inefficient agricultural practices (Greer, 2013; Kengyel, 2016). Nowadays, agriculture generates only around 1.3% of the EU's GDP, and represents around 4% of total employment, yet more than one-third of the EU budget is spent on the CAP. Nevertheless, its share of the EU budget has decreased very sharply over the past four decades, from 65.5% in 1980 to 35% in 2020 (European Commission, 2021c). These changes reflect the expansion of the EU's other responsibilities and cost savings from reforms. The CAP budget for 2014-2020 allocated €291.3 billion for direct payments (71.3% of the CAP budget), €17.5 billion for market interventions (4.3%) and €99.6 billion for rural development (24.4%). Agricultural expenditures totalled €408.3 billion from 2014 to 2020 (European Commission, 2021b). Figure 1 shows the gradual restructuring of EU level CAP expenditure and its GDP share.

According to the regulation on the MFF 2021-2027, €378.6 billion (at current prices) will be available to CAP beneficiaries, representing 31% of the total EU budget (Council of the European Union, 2020). Rural development measures under the CAP will be given additional resources from the Next Generation EU (NGEU) programme to fund economic and social recovery following the COVID-19 crisis (€8 billion).[2] Total CAP commitments for the 2021-2027 period are therefore €386.6 billion. The CAP's first pillar (financed by the European Agricultural Guarantee Fund) has a €291.1 billion budget. Up to €270 billion will be allocated to income support programmes, with the rest going to agricultural sector support (European Commission, 2021a). The overall allocation for the second pillar of the CAP (financed by the European Agricultural Fund for Rural Development) is €95.5 billion. This includes €8 billion from the NGEU recovery instrument. The CAP budget for 2021 accounts for 33.1% of the 2021 EU27 budget (€55.71 billion). As shown in Figure 2, direct payments and market measures (Pillar 1) represent 76.8% of agricultural appropriations (€40.4 billion) and rural development measures (Pillar 2) 23.2% (€15.3 billion).

2 Next Generation EU will increase the budget to assist rural areas in making the systemic improvements needed to meet the European Green Deal and digital transformation goals.

Figure 1
**The evolution of CAP expenditure**
current prices

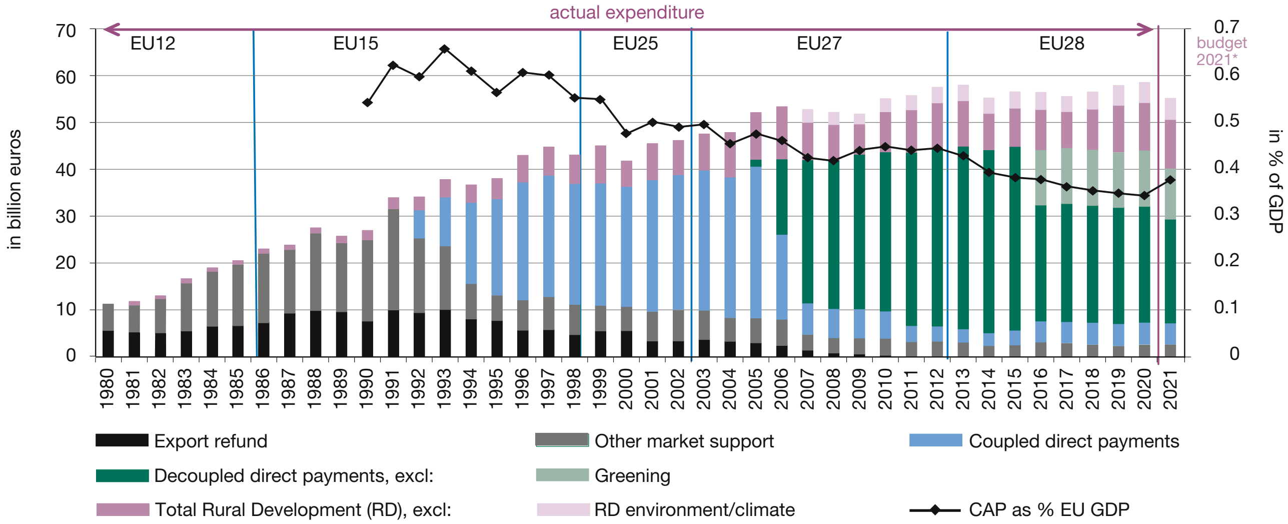

Note: 2021: budget amounts, coupled direct payments including POSEI and SAI direct payment component and Annex I Regulation 1305/2013.

Source: European Commission, 2021a.

## Renationalisation and co-financing as connected approaches

Although, the issue of renationalisation of the CAP seems to be a taboo subject, it is not a new feature. Even the MacSharry reforms of 1992 increased both the demand and the scope for renationalisation. The shift from market support to direct income payments increased the involvement of national authorities in the implementation phase. Theoretically, it is a crucial issue whether renationalisation of the CAP is an applicable way to implement a policy sensitive enough to national and regional needs and priorities (Niemi and Kola, 2005). It must be emphasised that there is no fixed framework for a renationalised agricultural policy yet, as negotiations about the topic have stalled or were neglected (Matthews, 2018, 2).

When talking about renationalisation of the CAP, we refer to a process of giving several competences from EU institutions back to the member states in terms of decision-making or financing or implementation – or all of these. In theory,

> renationalisation mainly deals with two issues: should Member States have more power and freedom on decisions of agricultural policy, and should there be a shift from common financing back to national funds? Renationalisation can be understood as a propensity to reinforce the power of the Member States in CAP matters at the expense of the EU decision-making process. (Niemi and Kola, 2005, 24)

What would be the specific effects of renationalisation of the decision-making processes and implementation? First of all, renationalisation would help national governments to become more sensitive and aware of regional needs. Member states would have more freedom in terms of decisions in the agricultural sector and more autonomy with the policies they would like to implement. The main reason why a more decentralised decision-making process is needed is that the CAP cannot handle the level of agricultural diversity in the EU. It is natural that there is a high level of diversity in terms of productivity, share in the economy, number of people employed, level of modernisation, etc. (Grochowska and Koisor, 2008).

With respect to the CAP, two principles that may be at stake in case of renationalisation are market unity and financial solidarity. There is a general impression that renationalisation could endanger the single market: If the time comes for national measures and financing, a single homogenous market is naturally harder to maintain. If CAP policies are no longer wholly financed from the EU budget but rather by member states, this would create the risk of divergence between states that are able and states that are unable to provide funding for these policies. This process would undermine the financial solidarity achieved by funding from the EU budget.

This divergence could in turn harm the principle of market unity, when the variation in financial capabilities between member states results in such a significant dispar-

Figure 2
**CAP allocations in the period 2021-2027**
commitments in current prices, billion euros

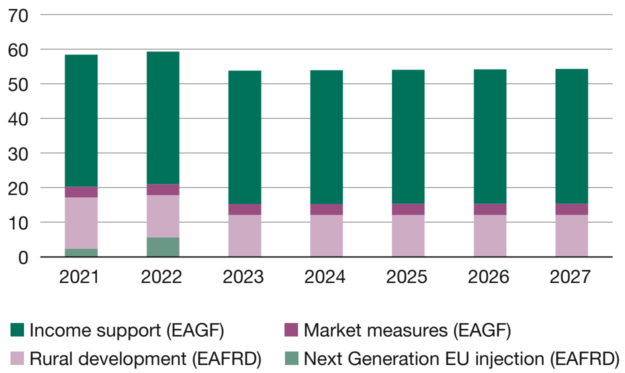

Note: EAGF: European agricultural guarantee fund, EAFRD: European agricultural fund for rural development.

Source: European Commission, 2021a.

ity in treatment of farmers in different member states that their relative competitiveness is affected by the nationally funded support measures.

> As a result, questions arise of how market unity, a fundamental principle of the CAP, may comply with steps towards more national influence… Renationalisation should not pave the way for governments to give unlimited national aids to their agricultural sectors. If market unity should be conserved along with a more nationally-oriented policy, it would be essential that the Commission should be able to survey – and enforce – that national assistance … do not give rise to unfair competition (Niemi and Kola, 2005, 37).

It is crucial that national policy should be in conformity with the EU's main rules, including the principle of the four freedoms and the rules defined by EU competition policy (including rules on state aids).

The core idea behind renationalisation and co-financing is the same: to take the burden off the EU's shoulders in terms of financing and to give a bigger role to member states. The main difference is that while renationalisation would change CAP into a policy that could turn the supranationalist CAP into a multi-layered system of regions and nationals, co-financing would mainly mean a financial redistribution. Therefore, co-financing does not aim to cause an in-depth change and spare the common market feature of CAP (Heinemann, 2017, 10). The rejection of co-financing is just the result of path dependency. Obviously, because of national budgetary interests, the member states would like to maintain the funding of agricultural policy from the EU level budget.

When we look at the proposed legal frameworks for the period 2023-2027, the main novelty is that each member state should prepare a national CAP Strategic Plan describing how to achieve the defined common objectives. This new approach gives member states more flexibility to take local conditions into account. Obviously, the new rules represent changes in the implementation of the policy; therefore, it can be stated that in some way the renationalisation is going to start – at least from the point of view of implementation. At the same time, it should be emphasised that the policy will be governed through common objectives and criteria and it will be monitored according to defined indicators. The new regulatory framework will avoid "transforming the CAP into an 'à la carte' agricultural policy which would then lead to its renationalisation" (Bourget, 2021, 2). On the other hand, co-financing will not become part of the new implementation model of the CAP.

## Potential advantages and disadvantages of co-financing

Practically speaking, co-financing should be introduced to address the huge burden of financing the CAP on the EU budget (Kengyel, 2016). As member states are obliged to co-finance Pillar 1, the EU could focus on different challenges. At the same time, it can be assumed that the ownership of the policy would be strengthened in the member states and it would result in a more efficient use of budgetary resources.

The main considerations in favour of the introduction of co-financing can be summed up as follows (Matthews, 2018; Heinemann, 2017; Heinemann and Weiss, 2018):

- The reduction of EU level agricultural subsidies through the possibility of national co-financing of Pillar 1 payments would be the only way to make a much better use of the very limited resources available at the EU level. A 30%-40% national co-financing rate would save annually about €15 billion to be spent on other EU policies.

- National co-financing of Pillar 1 would put the two pillars of the CAP on equal footing. This will result in a more balanced approach during the discussions about the roles of the two pillars.

- Theoretically, national co-financing of Pillar 1 payments could ensure continued transfers to farmers in the event of cuts in the EU CAP budget. However, it should be noted that the possibility of receiving additional funding from the national budgets could depend on the actual budgetary situation in a member state.

There are some possible cons for a co-financing system. However, these counter-arguments are mainly based on misleading assumptions that can be questioned. The counter-arguments can be summarised as follows:

- Member states often emphasise that Pillar 1 payments should be financed 100% from the EU budget as they implement common policy. This argument may sound nice, but it does not reflect reality. Other common policies of the EU are also co-financed by the member states. In fact, Pillar 1 of the CAP is an exception to the rule.

- There are fears that national co-financing would lead to an unbalanced distribution of resources if there are member states that have the necessary resources to provide high-level co-financing to farmers while others are unable to do this. Contrary to this argument, the experience of other EU policies that require national budgetary contributions shows that member states are able to find the required resources for co-financing.

- There is also an assumption that the introduction of co-financing would lead to the destructive race of national subsidies, which would distort the competition within the single market. With regards to this point, EU competition policy rules should be respected, which means that subsidies would remain under strict control.

## Concluding remarks

Based on the experiences of the past decades, a radical reform of EU level agricultural policy is needed due to the fact that, besides being extremely costly, the subsidy system has not proved very efficient. In addition, the Common Agricultural Policy is not able to take into account and handle the different needs and conditions of different member states. One way to renationalise the CAP would be to implement a policy sensitive enough to national and regional needs and priorities.

To conclude, we return to the original question: Are renationalisation and co-financing justified? First, we assume that co-financing of the CAP is justified, especially if we consider that member states already co-finance the CAP Pillar 2. It seems to be an appropriate answer to the changes happening in the EU today (new challenges and emerging priorities which require EU level budgetary resources). It would be a rational reform direction. Co-financing should, however, be strictly limited and should obviously fulfil EU competition rules in order to avoid the risk of distortion of competition and to secure a level playing field.

The renationalisation of regulatory frameworks should be more balanced. Renationalisation is less justified than co-financing in the context of climate-related and environmental issues. Even if it could be a useful method from the point of view of more effective implementation in general, there may be some doubts about its impact on achieving the desirable environmental goals because it would require a coherent, international action. We may have positive expectations for the new rules on spending a high share of CAP expenditures on climate policy-related goals and "eco-schemes" to support environmental and climate requirements in the period 2023-2027. However, increasing flexibility may have a negative impact on environmental ambitions in different member states and this should be avoided. There are fears that the new delivery and implementation frameworks starting from 2023 will provide a scope for enhanced flexibility that allows member states to choose low-ambition implementation pathways.

Concerning the current budgetary period, the member states, as usual, agreed to "national envelopes" for financing of the CAP. In practice, this approach can strengthen the impression of unconditional entitlement for funding. In addition, the Pillar 1 payments will continue to be financed fully from the EU budget, the option for introducing co-financing was neglected during the negotiations. There are doubts that even the conditionality for receiving the subsidies will not become more effective. If monitoring is going to concentrate much more on spending than on monitoring real performance-based impact indicators, the efficiency of using EU budgetary resources to support agricultural activities will definitely not improve in the future.
